# Detection of zoonotic nematodes in brown trout, *Salmo trutta,* an introduced popular edible freshwater fish in Australia

**DOI:** 10.1007/s00436-025-08533-w

**Published:** 2025-08-06

**Authors:** Shokoofeh Shamsi, Luke Pearce

**Affiliations:** 1 Gulbali Institute, School of Agriculture, Environment and Veterinary Sciences, Charles Sturt University, Wagga Wagga, Australia; 2 NSW Department of Primary Industries, Fisheries, Habitat & Threatened Species Unit, Freshwater Environment Branch, Albury, Australia

**Keywords:** Zoonotic nematodes, Freshwater fish, Foodborne parasites, Seafood, Food safety

## Abstract

Introduced freshwater fish species in Australia, such as brown trout (*Salmo trutta*), are commonly consumed and play a significant role in recreational fisheries. However, little is known about their potential to act as carriers of zoonotic parasites. This study investigated the presence of zoonotic nematodes in brown trout collected from above and below Winburndale Dam in New South Wales, Australia. Large nematodes were observed in the musculature during visual inspection. Additional nematodes were recovered following overnight incubation of the fish tissue and the gastrointestinal tract. Morphological and molecular analyses identified the large larvae in the muscle tissue as *Eustrongylides excisus*. In contrast, the gastrointestinal nematodes were identified as *Contracaecum bancrofti, Contracaecum rudolphii* D and one *Contracaecum* larva that may represent a previously undescribed species. Based on our findings, we recommend analysing the ITS-1 and ITS-2 regions separately when conducting BLAST analyses for species-level identification of *Contracaecum* larvae, as many early GenBank submissions contain only partial sequences. Of parasites found, *Eustrongylides* larvae were recovered from muscle and body cavity tissues, posing a direct food safety risk, while *Contracaecum* larvae were confined to the gastrointestinal tract and emerged only during post-mortem incubation, highlighting the value of supplementary observational techniques in detecting parasites that may otherwise be overlooked during routine dissection. The differences in parasite occurrence between upstream and downstream populations may be attributed to variations in diet, and water flow dynamics, particularly in relation to *Eustrongylides* infection. *Contracaecum rudolphii D* larvae, which had previously only been reported from marine fish, were found in a freshwater system for the first time in Australia. This study provides the first molecular confirmation of co-infection with multiple potentially zoonotic nematodes in brown trout in Australia, underscoring the need for parasite monitoring and proper food safety practices in freshwater fish species consumed by humans and their pets, such as cats and dogs.

## Introduction

Approximately, 43 species of introduced fish are recorded or established in inland freshwater systems across Australia (McKay 1984; Koehn and MacKenzie 2004). While these introduced species have drawn considerable attention for their adverse ecological impacts, such as competition and predation with native fauna, habitat alteration, and threats to biodiversity (Fletcher 1986; Arthington 1991; Keam 1994; Cadwallader 1996; Gillespie 2001; Tay et al. 2007), comparatively little research has been conducted on their role in food safety and public health. This oversight is notable, particularly as many of these species are important to recreational fisheries and, to a lesser extent, aquaculture. Among them, the brown trout (*Salmo trutta*) is widely distributed and frequently consumed (Jackson 1980; Anonymous 2017).

Brown trout, introduced from Europe in the late nineteenth century, have established stable populations throughout southeastern Australian rivers, lakes, and streams. It is a popular angling target (Ashburner 1978) and has, over time, become integrated into local ecosystems and human consumption patterns. While helminth surveys of *Salmo trutta* have been conducted extensively in parts of Europe, including recent detailed work in Spanish river systems (Ahmad et al. 2018; Couso-Pérez et al. 2023), such investigations remain limited in Australia. Despite its accessibility and appeal as an edible species, little is known about its potential role as a carriers of zoonotic parasites in Australia (Beumer et al. 1982).

Nematodes, with numerous zoonotic species, are among the most common and diverse parasitic helminths in fish. Of these, members of the family Anisakidae have received the most attention due to their zoonotic potential, particularly *Anisakis*, *Phocanema*, *Pseudoterranova* and *Contracaecum* spp., which can cause anisakidosis in humans following the consumption of raw or undercooked infected fish (Shamsi and Barton 2023). While the majority of such cases have been reported in marine systems, concern is growing about zoonotic nematodes in freshwater species as well (Poulin et al. 2011; Rusconi et al. 2022). An emerging threat in this context is *Eustrongylides* spp., an introduced nematode increasingly reported from Australian freshwater systems (Shamsi et al. 2023). Its life cycle involves aquatic oligochaetes as intermediate hosts and piscivorous birds as definitive hosts, with fish serving as second intermediate or paratenic hosts (Anderson 2000). The larval stages of *Eustrongylides* are capable of causing severe pathology in fish (Paperna 1974) and may pose a zoonotic risk to humans, especially when fish are consumed raw or inadequately cooked (Wittner et al. 1989; Eiras et al. 2018; Shamsi and Sheorey 2018).

The presence of zoonotic nematodes in edible freshwater fish species, particularly those that are widely consumed, can be concerning for public health, fisheries sustainability, and regulatory oversight. In Australia, research into the parasite fauna, especially zoonotic agents of introduced freshwater fish, remains limited. Existing literature is heavily skewed toward marine environments, leaving inland systems understudied. Furthermore, the effects of environmental and anthropogenic factors, such as habitat fragmentation and water regulation by dams, on parasite transmission dynamics are poorly understood. Therefore, this study aimed to investigate the occurrence of zoonotic nematodes in brown trout.

## Materials and methods

**Fig. 1:**
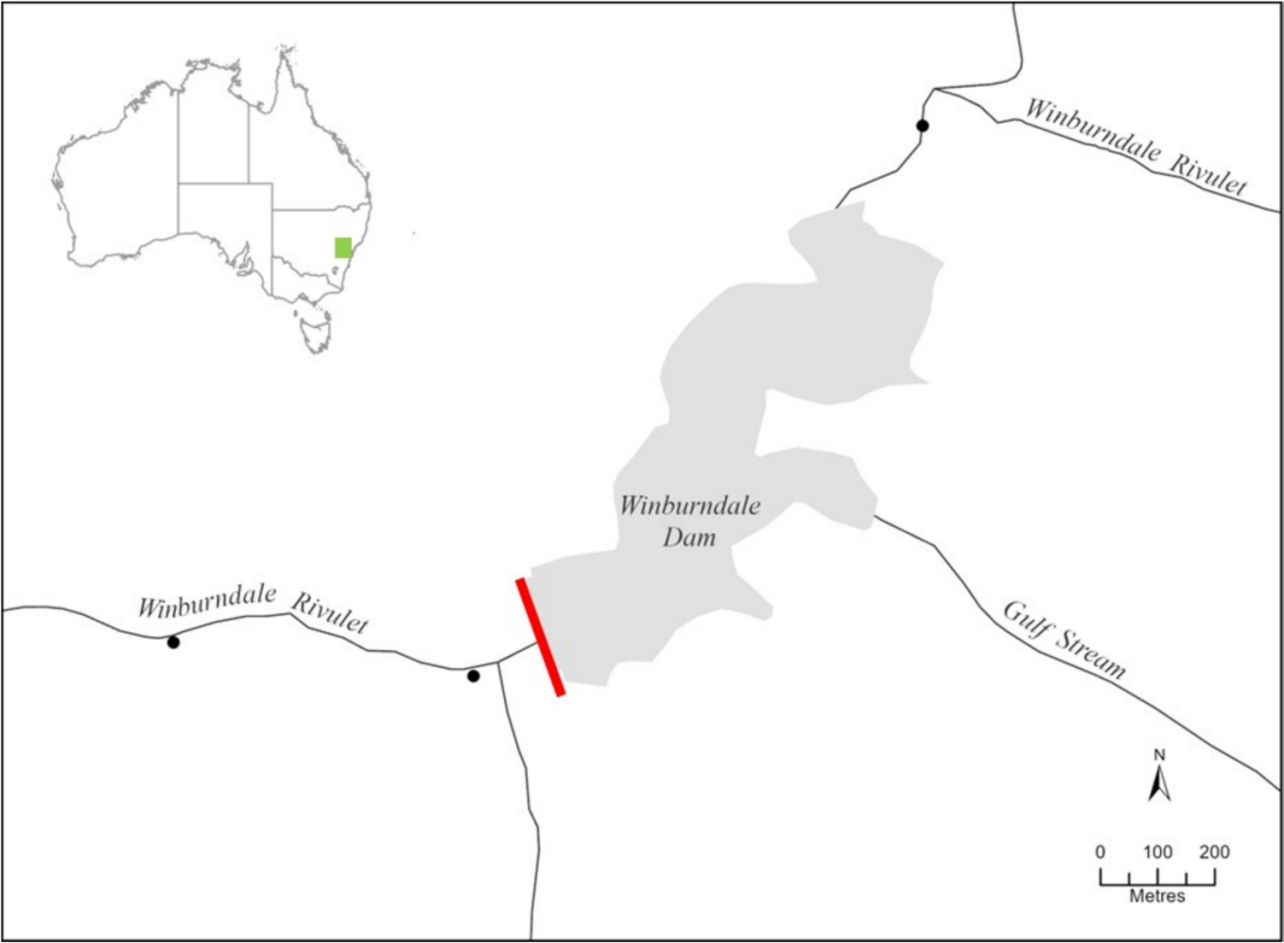
Map of the study area in New South Wales, Australia. The red line indicates the location of the Winburndale Dam wall. Black circles represent sampling sites where brown trout (*Salmo trutta*) were collected. The inset map in the top left corner shows the location of the river system and dam (green square) within the broader Australian context

A total of 40 brown trout (*Salmo trutta*) were submitted to Shamsi’s Parasitology Laboratory at Charles Sturt University. They were received dead and on ice. They were collected from a freshwater river system in New South Wales, Australia, on 17 February 2023. The brown trout were collected to investigate potential differences in parasite infection related to habitat variation associated with Winburndale Dam (Fig. 1) and also to examine their coexistence with other exotic species, such as carp and redfin perch below the dam but not above. Parasite specimens in the present study have been deposited in the helminth collection at the South Australian Museum under accession numbers: AHC 49587 (*Eustrongylides* larvae) and AHC 49588 (*Contracaecum* larvae).

Of the 40 brown trout submitted to the laboratory, 20 individuals were collected from upstream of the dam (five from the impounded waters of the dam itself, and the remaining came from the stream section directly above the dam), and 20 from downstream. Fish from above the dam were sampled from a combination of locations within the dam itself and a 150-m stretch of the stream extending upstream from the high-water mark of the dam (approximate central coordinate: −33.381636° 149.783062°). Fish were collected from below the dam between two defined points downstream (at −33.390251° 149.776002° and −33.389749°, 149.771280°).

Fish were examined for infection with nematodes following Shamsi and Suthar (2016), all organs, including the flesh of the fish, were thoroughly examined for presence of parasite both using visual examination and the incubation method. All nematodes were examined morphologically followed by obtaining sequences of the ITS and/or 18S of ribosomal DNA as described previously (Shamsi et al. 2023; Shamsi et al. 2024). The following epidemiological parameters were measured in this study: prevalence (P), mean abundance (MA), mean intensity (MI), and range (R) in accordance with (Bush et al. 1997).

## Results

**Fig. 2:**
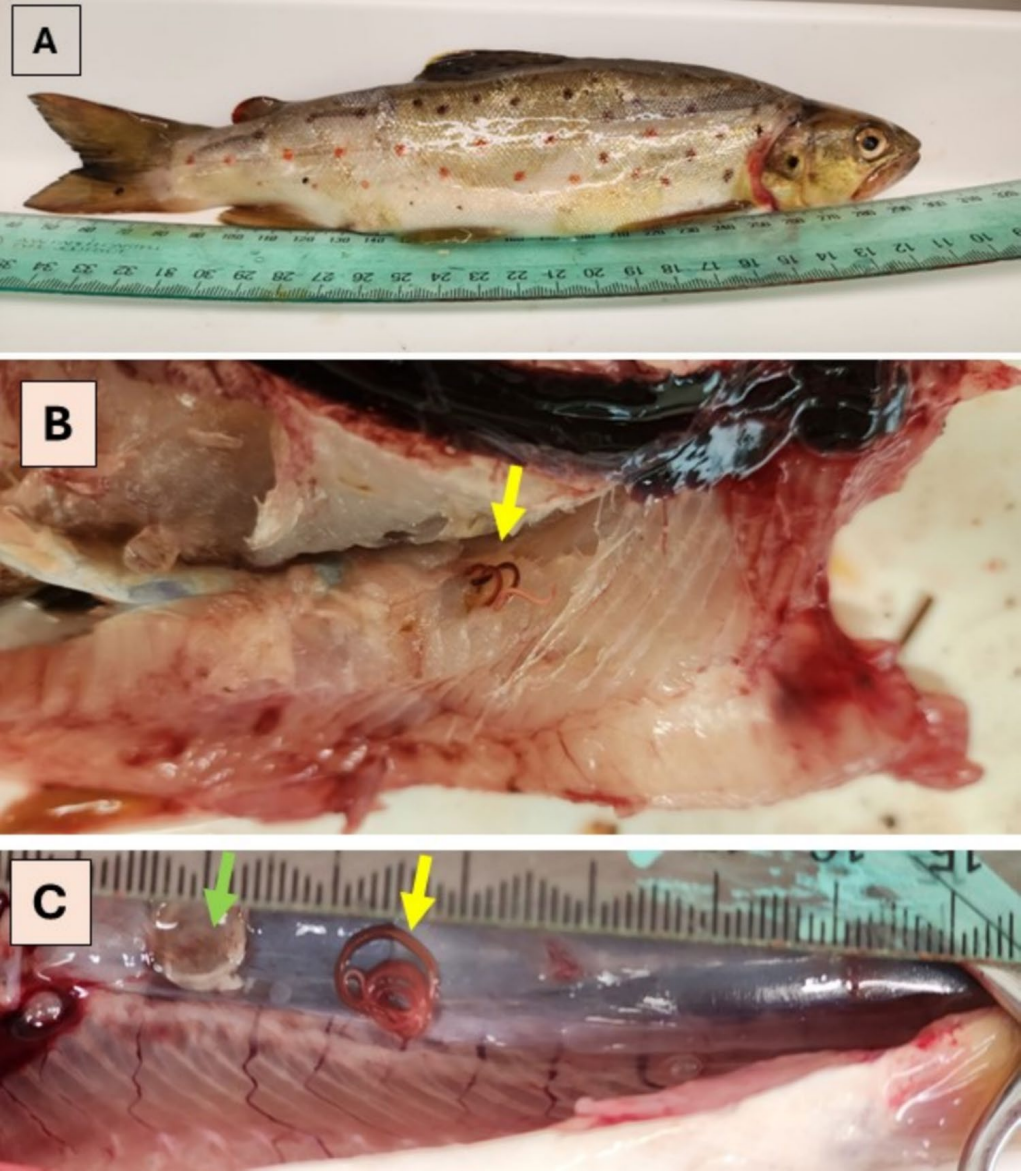
Images of an infected brown trout. (**A**) shows a fish that appears externally healthy; (**B**) shows a *Eustrongylides* larva (indicated by an arrow) deeply embedded in the muscle tissue; and (**C**) shows a larva (yellow arrow) removed from its cyst (green arrow). The cyst was located in the body cavity, attached near the spine and kidney, and had a colour similar to that of the adjacent tissue, making it difficult to detect for untrained eyes. A section of a ruler in millimetres is included for scale

**Fig. 3:**
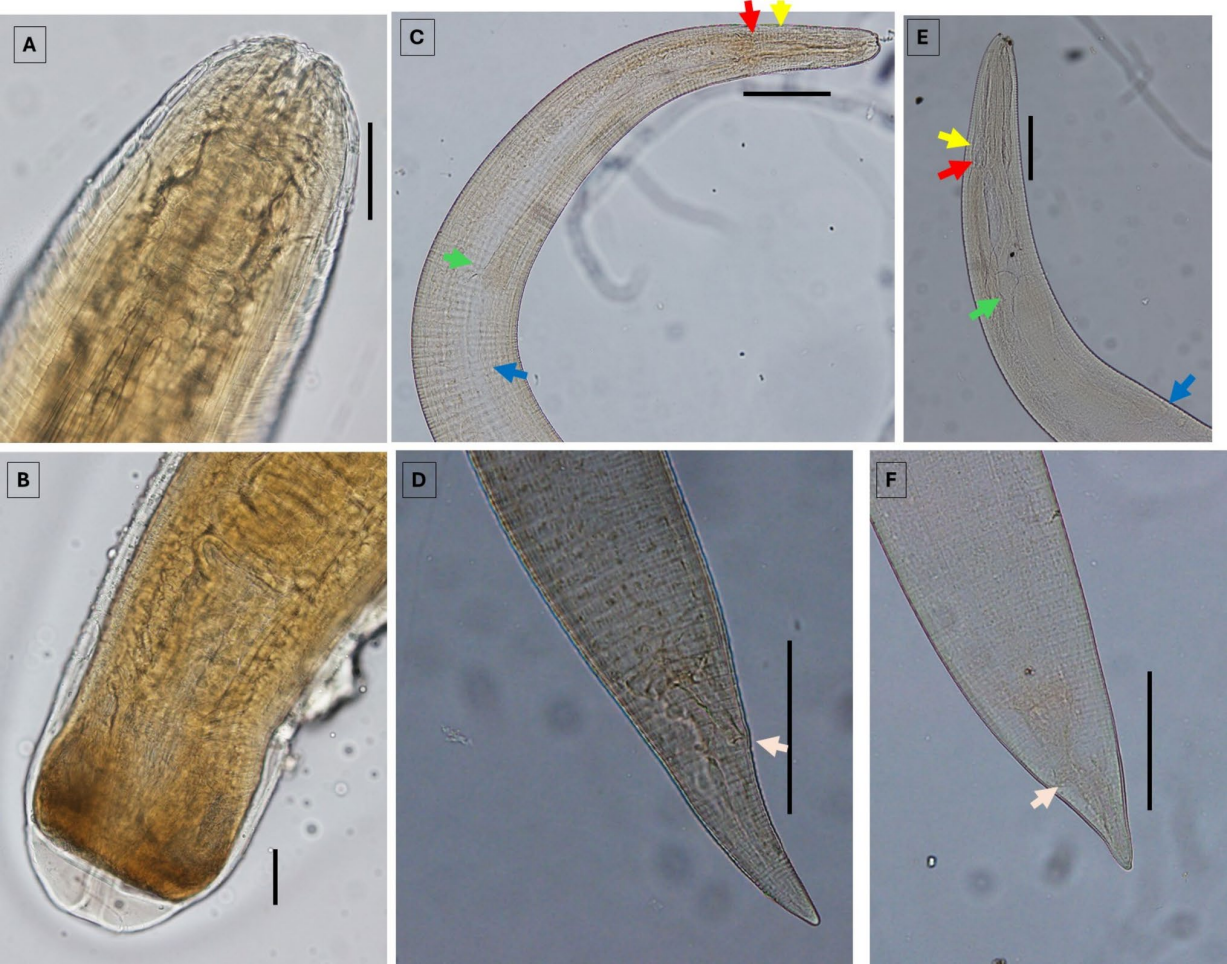
Microscopic images of the parasites found in the present study. (**A**) and (**B**) show the anterior and posterior ends of *Eustrongylides* larvae; (**C**) and (**D**) show the anterior and posterior ends of *Contracaecum bancrofti* larvae; and (**E**) and (**F**) show the anterior and posterior ends of *Contracaecum rudolphii D* larvae. Yellow arrows indicate the location of the nerve ring, red arrows show the location of the tip of the intestinal caecum, green arrows point to the location of the ventriculus, blue arrows indicate the tip of the ventricular appendix, and pink arrows show the location of the anus. Scale bars: 100 μ

Visual examination of the brown trout (Fig. 2A) revealed the presence of large red nematode larvae embedded within the muscle tissue and the body cavity (Fig. 2B and C). Additional larvae emerged from muscle samples following overnight incubation. Microscopic nematodes were also recovered from the gastrointestinal tract of the examined fish following overnight incubation. All nematodes recovered from the examined fish were identified as third-stage larvae (L3). Morphological examination revealed that they belonged to two genera: *Eustrongylides* and *Contracaecum* (Fig. 3).

Molecular analysis of the 18S rDNA sequences confirmed that the *Eustrongylides* larvae belonged to *Eustrongylides excisus*, as our specimens (GenBank accession nos. PV871169 to PV871172) showed100% similarity with sequences from well-identified adult specimens held in museum collections (South Australian Museum, Adelaide (Accession numbers: AHC 49214); GenBank accession no. ON209259; (Shamsi et al. 2023)). Larvae of *Eustrongylides* were found either embedded in the musculature or free and/or encysted within the body cavity of the fish (Fig. 2).

*Contracaecum* larvae were exclusively found embedded in the gastrointestinal tract. These were only detected following overnight incubation of the dissected digestive tracts at room temperature, which facilitated larval emergence. Molecular identification revealed the presence of the following *Contracaecum* species, based on a 100% identical sequence with well-characterised adult specimens in GenBank: *C. bancrofti* (GenBank accession no. PV893030 to PV893030), which matched GenBank accession no. OP782836 (Caffara et al. 2023), corresponding to adult *C. bancrofti* from the Australian pelican; and *C. rudolphii* D (GenBank accession no. PV893032 to PV893033), which showed identical sequences to GenBank accession nos. FM210251 (ITS-1) and FM210261 (ITS-2), derived from adult *C. rudolphii* D collected from the great cormorant (*Phalacrocorax carbo*) (Shamsi et al. 2009). It is noteworthy that when conducting BLAST analyses of the ITS region, the ITS-1 and ITS-2 regions should be analysed separately, as many early reports of *Contracaecum* species predate the widespread availability of long-read sequencing technologies.

Additionally, one unidentified species of *Contracaecum* was detected (GenBank accession no. PV893034). No identical sequence was available in the GenBank for partial or complete ITS region. The closest GenBank matches for this isolate were MW315554 for ITS-1 (Suthar and Shamsi 2021), and AY821753 for ITS-2 (Nadler et al. 2005), and AY821750 and OR985046 for the complete ITS region (Nadler et al. 2005; Dezfuli et al. 2024).

Table 1 provides a detailed summary of parasite prevalence, mean intensity, and abundance across fish specimens and locations. Notably, *C. bancrofti* was only detected in fish from above the dam, *Contracaecum* sp. was found only in fish from below the dam, while *C. rudolphii* D occurred in fish from both locations.

**Table 1:** Detailed information about the prevalence of infected fish and abundance of the nematode larvae found in the present study

Fish species	Location	No of fish examined	Nematoda (Total)		*Contracaecum* larvae		*Eustrongylides* larva	
			No of infected fish (P)	No of nematodes (MA, MI, R)	No of infected fish (P)	No of larvae (MA, MI, R)	No of infected fish (P)	No of larvae (MA, MI, R)
Brown trout	Below the dam	20	9 (45.0 ± 11.1)	20 (1.0 ± 1.3, 2.2 ± 1.1, 1–4)	7 (35.0 ± 10.7)	12 (0.6 ± 0.9, 1.7 ± 0.5, 1–2)	2 (10.0 ± 6.7)	8 (0.4 ± 1.2, 4.0 ± 0.0, 4–4)
Brown trout	Above the dam	20	10 (50.0 ± 11.2)	32 (1.6 ± 2.1, 3.2 ± 1.9, 1–7)	1 (5.0 ± 4.9)	2 (0.1 ± 0.4, 2.0 ± 0.0, 2–2)	9 (45.0 ± 11.1)	30 (1.5 ± 2.1, 3.3 ± 2.0, 1–7)
Total	All	40	19 (47.5 ± 7.9)	51 (1.3 ± 1.8, 2.7 ± 1.6, 1–7)	8 (20.0 ± 6.3)	14 (0.4 ± 0.7, 1.8 ± 0.5, 1–2)	11 (27.5 ± 7.1)	38 (0.95 ± 1.8, 3.4 ± 1.8, 1–7)

Abbreviations are as follows: *P* Prevalence; *MA* Mean Abundance; *MI* Mean Intensity; *R* Range

Brown trout from above the dam showed higher infection with *Eustrongylides* larvae compared to those from below the dam, both in terms of prevalence and the number of parasites per infected fish (mean intensity and abundance). In contrast, a greater number of brown trout from below the dam were infected with *Contracaecum* larvae.

Although no formal dietary analysis was conducted, visual inspection of stomach contents revealed a marked difference in the diet of brown trout from the two locations. Fish collected from above the dam predominantly had stomachs filled with small freshwater prawns. In contrast, those collected from below the dam had stomach contents consisting mostly of aquatic beetles and other insect larvae.

## Discussion

One of the key significances of the present study is the detection of the infectious larval stages of two zoonotic nematode genera, *Eustrongylides* and *Contracaecum*, in a popular edible fish species in Australia. There are numerous reports of human infection with these parasites globally. Human infections with *Eustrongylides* spp. have been reported in the United States and Sudan, causing gastrointestinal symptoms such as gastritis and, in severe cases, intestinal perforation (Guerin et al. 1982; Gunby 1982; Wittner et al. 1989; Narr et al. 1996; Eberhard and Ruiz-Tiben 2014). Human infections with *Contracaecum* spp. have been documented in Australia, the Baltic region and Japan (Schaum and Muller 1967; Shamsi and Butcher 2011; Nagasawa 2012).

Of the two zoonotic parasites identified, *Eustrongylides* spp. clearly pose a higher risk of human infection (Di Maggio et al. 2024) when fish is consumed raw or undercooked, as the larvae are primarily located in the edible musculature. In contrast, *Contracaecum* larvae are typically confined to the gastrointestinal tract and present a lower immediate risk to consumers. However, if infected fish are not gutted promptly, the larvae may migrate from the digestive tract into the muscle tissue, particularly during storage and handling (Smith 1999). Therefore, *Contracaecum* spp. represent a potential food safety concern primarily within the context of the food supply chain.

It is also important to recognise that the zoonotic potential of *Contracaecum* species is likely to vary depending on their definitive hosts and ecological traits. Some species, such as *C. osculatum*, are known to infect pinnipeds and have been implicated in zoonotic infections, while others are more commonly associated with piscivorous birds and generally assumed to pose a lower risk to humans. However, this assumption may in part reflect the lack of precise species-level identification in many earlier reports, rather than definitive evidence of lower zoonotic potential. In the present study, we identified *C. bancrofti* and *C. rudolphii D*, both of which are bird-associated species (Shamsi et al. 2009, 2019). Although human infections with bird-associated *Contracaecum* species seem to be rare, they cannot be entirely ruled out, particularly if post-harvest handling permits larval migration into muscle tissue, where they may be inadvertently consumed.

Moreover, as highlighted by Shamsi and Barton (2023), a significant number of published reports on anisakid larvae rely on assumed identifications rather than providing robust morphological or molecular evidence. This underscores the need for thorough and accurate species-level identification to better understand the public health risks posed by different *Contracaecum* species. Further studies on the infectivity and survivability of these parasites in humans are warranted to refine risk assessments and inform food safety practices.

In terms of specific identification of larval nematodes, morphological examination is generally limited to the genus level (Fagerholm 1991); therefore, molecular analyses of genetic sequences are essential for accurate species-level identification (Orecchia et al. 1986; Shamsi et al. 2011).

To date, the only nematode reported from brown trout (*Salmo trutta*) in Australia is *Eustrongylides*. *Eustrongylides excisus* has previously been recorded under various names, including *E. gadopsis*, *E. phalacrocoracis*, and *Eustrongylides* sp., based on older reports (Beumer et al. 1982). These names are now regarded as scientifically invalid (Measures 1988). The presence of *E. excisus* in Australia has since been confirmed both morphologically and molecularly (Shamsi et al. 2023). The sequence data obtained in the present study further confirm that *E. excisus* also infects brown trout in Australia. These findings provide necessary clarification regarding the taxonomic status of *Eustrongylides* larvae reported in earlier studies and verify their identity as *E. excisus*.

*Contracaecum bancrofti* larvae have previously been reported from a range of freshwater fish species in Australia, including bony bream (*Nematalosa erebi*, family Clupeidae), oriental weatherloach (*Misgurnus anguillicaudatus*, family Cobitidae), goldfish (*Carassius auratus*) and common carp (*Cyprinus carpio*, both family Cyprinidae), carp gudgeons (*Hypseleotris* sp., family Eleotridae), Murray–Darling rainbowfish (*Melanotaenia fluviatilis*, family Melanotaeniidae), eastern gambusia (*Gambusia holbrooki*, family Poeciliidae), and Australian smelt (*Retropinna semoni*, family Retropinnidae) (Shamsi et al. 2019). Among these, goldfish, common carp, and gambusia are introduced species in Australia (Lintermans 2004). The present study adds another introduced fish species, brown trout (*Salmo trutta*) to the list of hosts harbouring larval stages of *Contracaecum bancrofti*, a native Australian *Contracaecum* species.

The larval stage of *Contracaecum rudolphii* D has previously been reported from a marine fish, the flathead (*Platycephalus laevigatus*) (Shamsi et al. 2011). The present study represents the first report of this species from a freshwater fish. The presence of a *Contracaecum* larva with no identical sequence match in GenBank suggests the existence of a potentially undescribed species, for which the adult form has yet to be discovered and formally characterised.

When conducting BLAST analyses for species-level identification of *Contracaecum* larvae, it is essential to search the ITS-1 and ITS-2 regions separately. Many early GenBank submissions predate the widespread use of long-read sequencing technologies and often include only partial sequences corresponding to either ITS-1 or ITS-2. As a result, relying solely on full-length ITS sequences may overlook relevant, well-annotated records, particularly those linked to morphologically verified adult specimens. By analysing ITS-1 and ITS-2 separately, researchers can achieve greater resolution in species identification, improve taxonomic clarity, and avoid misinterpretation due to incomplete sequence data.

All parasite species identified in the present study use birds as their definitive hosts (Barus et al. 1978; Fagerholm 1998; Mazzone et al. 2019) and various aquatic invertebrates as their first intermediate hosts (Sprent 1954; Huizinga 1967; Anderson 2000; Dziekonska-Rynko and Rokicki 2007). Although the design of this study does not allow for robust statistical comparisons, the observed differences in parasite abundance and species composition between trout populations above and below the dam are noteworthy and warrant further investigation. Several environmental and ecological factors, including variations in water flow, habitat structure, and the composition of the fish community may influence these differences.

The section of stream above the dam, where the upstream fish were collected, is relatively unmodified and flows through native eucalypt forest, with riparian vegetation dominated by River She-oak (*Casuarina cunninghamiana*). The stream is generally shallow (mostly < 1 m), with a cobble and gravel substrate, submerged macrophytes, and structurally complex habitats such as woody debris, roots, and undercut banks. Apart from trout, the only other introduced fish species in this section is goldfish (Lintermans 2023; Annonymous 2012), which occur in the dam itself but not in the stream immediately above. The upper 7 km of this stream segment remain largely intact, although the area beyond this point becomes increasingly modified, featuring extensive pine plantations and the proliferation of exotic weeds. In contrast, the stream below the dam is broader and deeper, with some sections exceeding 1 m in depth. While gravel and cobble substrates are still present, parts of the stream also exhibit sandy or muddy bottoms. The riparian vegetation still includes River She-oak but is more heavily impacted by introduced grasses and weeds. Structural habitat is more limited, with a noticeable absence of submerged macrophytes and less woody debris. A significant presence of iron floc or iron-oxidising bacteria was also observed, potentially resulting from the release of water from lower layers of the dam. The fish community below the dam is markedly different, with the presence of additional introduced species such as carp and redfin, which are absent upstream. Flow regimes are also altered below the dam, being regulated and generally slower than the more natural flows upstream. The interplay of these ecological differences may significantly influence the dynamics of parasite transmission. For instance, slower flows and different substrate types downstream may facilitate the persistence of intermediate hosts or create favourable conditions for parasite larvae. Moreover, the presence of additional fish species, such as carp and redfin, may serve as alternative or amplifying hosts (Shamsi et al. 2021), potentially increasing transmission opportunities for parasites with broad host specificity.

The observed differences in parasite prevalence may also reflect variation in the presence and distribution of other vertebrate and invertebrate species in the region, which act as intermediate or paratenic hosts (Shamsi et al. 2021; Oliveira et al. 2024). This is particularly relevant given that the larval stages of both *Eustrongylides* and *Contracaecum* species are capable of infecting a wide range of aquatic and semi-aquatic animals, including fish, reptiles and amphibians (Yermolenko et al. 2021; Campião et al. 2024; Di Maggio et al. 2024; Oliveira et al. 2024; Cárdenas et al. 2025). Future studies incorporating broader ecological data and quantitative sampling will be important to clarify the mechanisms driving these differences in parasite transmission and distribution.

Future research should also consider the potential for interactions between co-infecting parasite species, which may affect infection success and disease outcomes. Gopko et al. (2018) experimentally demonstrated that the timing and sequence of infections significantly influence parasite establishment in *Salmo trutta*, highlighting the need to explore such dynamics in natural and semi-natural systems, particularly where fish host multiple parasite taxa.

In terms of parasite detection in fish, overnight incubation of gastrointestinal tissues enabled the recovery of *Contracaecum* larvae that were not visible during routine dissection. The internationally recognised techniques for quantitative assessment of anisakid larvae in fish musculature are the artificial digestion and UV-press methods (Anonymous 2021). The lack of standardised detection may result in either under- or over-estimation of true infection prevalence or intensity. Nevertheless, the use of exploratory methods can still provide valuable insights into parasite presence, particularly for species not yet well-studied in Australian freshwater systems.

In conclusion, this study provides the first molecular confirmation of co-infection with *Eustrongylides excisus*, *Contracaecum bancrofti*, and *Contracaecum rudolphii* D and an unknown *Contracaecum* parasite in brown trout in Australia. The findings highlight the zoonotic potential of these nematodes and emphasize the importance of handling and preparing freshwater fish intended for human consumption with care. Given the capacity of these parasites to infect humans through consumption of raw or undercooked fish, and the risk of larval migration during storage, these results highlight the importance of parasite monitoring and proper food safety practices throughout the supply chain.

In light of the zoonotic parasites identified in this study, particularly the presence of *Eustrongylides* larvae in edible muscle tissue, public health authorities may consider issuing or reinforcing specific guidelines for freshwater fish consumption. Concrete recommendations that could be communicated to consumers include avoiding consuming raw or undercooked freshwater fish, particularly from recreational fisheries, where inspection and control measures are limited; gutting fish immediately after capture to prevent potential migration of larvae from the gastrointestinal tract into the musculature during storage; freezing fish at –20 °C for at least 7 days or cooking thoroughly (to an internal temperature of ≥ 63 °C) to kill any infective larvae present; raising awareness among recreational fishers and consumers about the potential risks of zoonotic parasites and the importance of proper handling and preparation. These actions are consistent with food safety standards recommended by agencies such as Australian Food Safety Information Council (https://www.foodsafety.asn.au/topic/seafood-parasites/) the Food and Agriculture Organization (FAO), World Health Organization (WHO), and Codex Alimentarius (Food and Agriculture Organization of the United Nations 2014; Codex Alimentarius CAC/GL 88–2016 2016). Furthermore, incorporating parasite surveillance into local food safety protocols for freshwater fish, especially in regions where wild-caught fish are sold or shared, would improve early detection and risk mitigation.

## Data Availability

No datasets were generated or analysed during the current study.
